# Mental health policy and development in Egypt - integrating mental health into health sector reforms 2001-9

**DOI:** 10.1186/1752-4458-4-17

**Published:** 2010-06-24

**Authors:** Rachel Jenkins, Ahmed Heshmat, Nasser Loza, Inkeri Siekkonen, Eman Sorour

**Affiliations:** 1 Department of Health Services and Population Research, Institute of Psychiatry, Kings College London, De Crespigny Park, London, UK; 2 Department of Health Management, Health Care International (HCI), 35, Hadayek El-Obour Buildings, Salah Salem Road, Cairo, Egypt; 3 Ministry of Health, 3 Magles El Shaab Street, Cairo, Egypt

## Abstract

**Background:**

Following a situation appraisal in 2001, a six year mental health reform programme (Egymen) 2002-7 was initiated by an Egyptian-Finnish bilateral aid project at the request of a former Egyptian minister of health, and the work was incorporated directly into the Ministry of Health and Population from 2007 onwards. This paper describes the aims, methodology and implementation of the mental health reforms and mental health policy in Egypt 2002-2009.

**Methods:**

A multi-faceted and comprehensive programme which combined situation appraisal to inform planning; establishment of a health sector system for coordination, supervision and training of each level (national, governorate, district and primary care); development workshops; production of toolkits, development of guidelines and standards; encouragement of intersectoral liaison at each level; integration of mental health into health management systems; and dedicated efforts to improve forensic services, rehabilitation services, and child psychiatry services.

**Results:**

The project has achieved detailed situation appraisal, epidemiological needs assessment, inclusion of mental health into the health sector reform plans, and into the National Package of Essential Health Interventions, mental health masterplan (policy guidelines) to accompany the general health policy, updated Egyptian mental health legislation, Code of Practice, adaptation of the WHO primary care guidelines, primary care training, construction of a quality system of roles and responsibilities, availability of medicines at primary care level, public education about mental health, and a research programme to inform future developments. Intersectoral liaison with education, social welfare, police and prisons at national level is underway, but has not yet been established for governorate and district levels, nor mental health training for police, prison staff and teachers.

**Conclusions:**

The bilateral collaboration programme initiated a reform programme which has been sustained beyond the end of the funding. The project has demonstrated the importance of using a multi-faceted and comprehensive programme to promote sustainable system change, key elements of which include a focus on the use of rapid appropriate treatment at primary care level, strengthening the referral system, interministerial and intersectoral liaison, rehabilitation, and media work to mobilize community engagement.

## Background

Although most donor and development agency attention has hitherto been focussed on communicable diseases in Egypt, the importance of non-communicable diseases has recently been recognised and in 2000, Egypt established the Epidemiology and Surveillance Unit to track the incidence of noncommunicable diseases in Egypt , especially diabetes and cardiovascular disease, but it did not include mental health which is important in its own right [1] and in its influence on health, education and social goals [2].

Substantial and enduring improvements in mental health services require an integrated policy and strategy, including systematic educational interventions to equip service providers with necessary knowledge and skills, public education to raise awareness of the importance of mental health for everyone, and of the issues surrounding mental disorders in the community, combined with organisational reforms to enable interventions to be embedded in the health system and in routine care [3,4]. A previous significant WHO initiative in Alexandria, although successful in the short term, did not continue or disseminate sustainably across the country after the donor funding came to an end [5].

The project described in this paper sought to meet these challenges, aiming to introduce sustainable mental health policy and decentralised development across the country. Therefore this paper describes an integrated approach to mental health policy development in Egypt, firstly funded by a Finnish bilateral aid project 2000-7, and then by the Ministry of Health and Population (MOHP) 2007- present, with sustained technical support from the WHO Collaborating Centre (WHOCC), Institute of Psychiatry, and from WHO Eastern Mediterannean Regional Office ( EMRO) and WHO Geneva. The policy objectives of the programme were fourfold: firstly to conduct detailed situation appraisal of mental health within Egypt; secondly to use that information to develop appropriate and integrated mental health policy and plans; thirdly to develop mechanisms for sustainable implementation of policy across the country, using locally available resources and integrated into local systems; and fourthly to monitor and evaluate progress to fine tune implementation.

## Methods

A multi-faceted and comprehensive programme which combined situation appraisal to inform planning; establishment of a health sector system for coordination, supervision and training of staff at each level (national, governorate district and primary care); development workshops; production of toolkits, development of guidelines and standards; encouragement of intersectoral liaison at national, governorate, district and local levels; public education; and integration of mental health into health management systems, and specific efforts to improve forensic services, rehabilitation services, child psychiatry services.

### Country situation appraisal

Situation appraisal was conducted briefly first in 2001, and then in more detail in 2003-4: firstly by identification and analysis of national and local data and ministry and other documents; secondly by site visits to relevant sectors - health, education, social welfare, police, prisons, non-governmental organisations (NGOs) at national, governorate, district and primary care levels, accompanied by detailed consultation and discussion with professionals, clients, families and other stakeholders, thirdly by workshops spearheaded by the MOHP fourthly using the information collected above to construct a mental health country profile of Egypt - a structured systematic assessment of context, needs, resources, provision and outcomes, containing qualitative and quantitative data, and compiled by multiple stakeholders [6,7]; and finally an epidemiological survey reported elsewhere [8].

### National policy dialogue

The authors undertook sustained policy dialogue on mental health with the Ministry of Health and Population and other key ministries about both generic and specific issues. The generic issues included firstly the need for national mental health policy; secondly the need for a strengthened mental health section within the Ministry, in order both to represent mental health in generic policy development and to coordinate mental health for Egypt; and thirdly the need to integrate mental health into generic health sector reforms.

The specific policy dialogue comprised consideration of the needs, strengths and challenges identified in the situation appraisal. This guided the development of written policy and strategic action plans to address Egypt's needs in the light of Egypt's context and resources. Further specific policy issues addressed included preparation of updated mental health legislation and executive regulations for the legislation.

### Organisational and operational interventions

The mental health programme has been planned and implemented within the organisational context of the Egypt Health Sector Strategy which is committed to universality, quality, equity, efficiency and sustainability of health and health care, and which has had an extensive programme of health sector reforms since 2000 focussing on the establishment of a holistic family health approach, decentralization of MOHP specialist service provision and management to the district level, integration of service provision at the facility level through the family physician system (with the intention that no-one should live further than 2 km from the nearest health facility), and the creation of organizational structures, effective management systems, building competent capabilities, regulatory framework and institutional relationships that enhance and sustain the reform of the Egyptian Health Sector. Thus the time was right for mental health reforms.

Other organisational and operational interventions, designed in collaboration with the MOHP and iteratively modified during stakeholder consultation workshops, included establishment of the Section (Department) of Mental Health, within the Central Administration of Curative Care Services, in the MOHP; gathering and analysing data submitted by the governorates, and disseminating supportive information from the MOHP; inclusion of mental health in health management information systems; guidelines for roles and responsibilities on mental health within the various tiers of the overall health service together with delineation of the potential contributions of other key sectors; distribution of good practice clinical guidelines; establishment of systems for continuing professional development for primary care; educational training of trainers so they can deliver training on mental health for front line primary care staff; and establishment of a system of coordination and supervision of primary care by governorate coordinators.

### Primary Care training

The Egymen project developed plans in 2003 to train governorate trainers and supervisors to support primary care, in a staged manner in five pilot governorates which were also the focus of the Egypt health sector reform programme. The training consisted of theoretical presentations, discussions and role plays to rehearse skills and competencies. Trainers were trained who then rolled out the training across five governorates, and regular systematic supervision was delivered.

A toolkit of training materials was developed for the continuing professional education of primary care workers which includes a training guide and role play guides, videos and teaching slides. The training courses were conducted simultaneously for doctors, nurses and social workers to enhance their work as a team. The training content aimed to empower the professional roles of each cadre. The emphasis for doctors' training was early detection and prevention of people with mental illness in the community, while the nurse training focussed on understanding mental illnesses, their causes and consequences, and how to carry out the nurses' supportive role in the care of mental patients. Social workers were encouraged to add mental health related topics in their health education sessions as well as to screen mental problems at home visits.

The duration of doctors' training was six days, while the nurse and social worker training lasted four days. The last day was a team day, where doctors, nurses and social workers jointly discussed, diagnosed and prepared a management plan for a written patient case vignette; each of them contributing their special knowledge and expertise about the case.

After a training course the trainees were provided with a follow up support: At the end of a theoretical course the trainees were given logbooks for taking notes and for data collections of mental cases. These materials were reviewed by consultant psychiatrists during their regular field supervision visits. This support was provided to each primary health care (PHC) unit monthly, for six times over a six month period immediately after a training course. The whole trained team attended these supervisory consultations. Either clients with mental illness were present during a supervisory consultation or mental cases were reviewed from logbooks.

Data on service utilisation, routinely collected by the MOHP, was used to evaluate progress, augmented by qualitative information from workshops and focus group discussions to discuss key themes and issues emerging from the implementation of the work.

### Specialist training

The Egymen project also recruited expert assistance to capacity build specialist expertise and develop services for forensic psychiatry, rehabilitation and child psychiatry, and continued support was given by the Finnish government, 2000-9, WHO Collaborating centre, Institute of Psychiatry, London 2000-9 and the RCPsych 2006-9. This comprised visits to Egypt by Finnish and UK experts, Egyptian study tours to Finland, England and other European countries, and specific tailored placements in the UK. Funding for service developments from the Finnish government, and the MOHP has continued to access expert assistance from the UK for forensic psychiatry and legislation.

### Good practice guidelines

The good practice guidelines for primary care in Egypt were adapted from the WHO PHC mental health guidelines in a multistage process. An initial workshop was held in Cairo to do a first stage adaptation with Egyptian colleagues to tailor the guidelines to a middle income country setting in 2003. The second stage of adaptation comprised a questionnaire which was sent to senior psychiatrists and psychiatric nurses to ask their views of the draft and suggestions for improvement. In the light of the replies, a second draft was produced which was extensively discussed in a workshop in Cairo in 2004, revised, and then translated into Arabic, back translated and improved. The guidelines have been distributed to all primary care staff as they are receiving training in mental health 2004-7.

## Results

### Results of the situation appraisal and ensuing interventions

Additional file 1 summarises the key integrated findings of the situation appraisal, derived from the documentary analysis, the site visits and consultations with the various sectors and various tiers of the health service, the regional workshops, and the construction of the mental health country profile for Egypt which may be accessed on http://www.mental-neurological-health.net/[7]. The fifth method of situation appraisal, the epidemiological study, conducted by others, is reported elsewhere [8].

### Contextual factors

**Figure 1:**
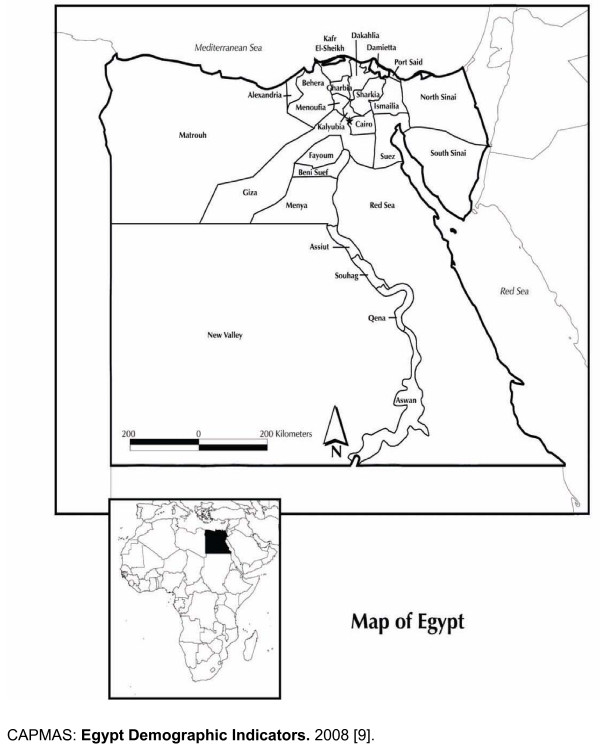
**Map of Egypt showing the governorates**.

Egypt is located in the northeast corner of the African continent (see Figure 1). It is bordered by Libya to the west, Sudan to the south, the Red Sea to the east, and the Mediterranean Sea to the north.

Egypt has the largest, most densely settled population among the Arab countries. The total area of the country covers approximately one million square kilometres, but much of the land is desert. Egypt is administratively divided into 26 governorates (regions) and Luxor City, and 203 districts. The four Urban Governorates (Cairo, Alexandria, Port Said, and Suez) have no rural population. Each of the other 22 governorates is subdivided into urban and rural areas. Nine of these governorates are located in the Nile Delta (Lower Egypt), eight are located in the Nile Valley (Upper Egypt), and the remaining five Frontier Governorates are located on the eastern and western boundaries of Egypt (see Figure 1.)

Egypt is a middle income country with a gross national income of 1580 USD per capita, compared with the regional average of 2794USD for Middle East and North Africa as a whole. The latest population census in Egypt was carried out in November 2006 indicating a de facto population of 72.2 million. This number excludes the roughly 3.9 million Egyptians who are living abroad. By the beginning of 2008, it is estimated that population had increased by around one and half million to reach 74.3 million [9]. 43% of the population is living in urban areas, with 20 million in Cairo (as a whole) and 4.2 million in Alexandria. The remainder of the population is largely concentrated along the Nile, with poor transport to other areas of the country. Life expectancy at birth is 71 years, infant mortality 2.9%, child malnutrition 5%, Maternal mortality: 55/100,000 birth (2008) (compared with 75/100,000 birth in 2002) and 79% of births are attended by skilled health workers and .98% of the population have access to clean water and literacy is 71% [10,11]. Egypt has 203,100 doctors altogether which is on average 1 per 370 population but there is maldistribution between urban and rural areas, and a large proportion operate exclusively in the private sector.

60% of deaths are from communicable diseases. Malaria and polio have essentially been eradicated, but schistosomiasis and leishmaniasis remain prevalent, accompanied by a very high prevalence of active hepatitis C, (10-15% of population are carrying hepatitis antibodies and at least 5 million have the active virus), largely caused by the use of inadequately sterilized needles in early treatment campaigns for schistosomiasis in the 1980s, and more recently due to increased kidney dysfunction and increase use of inadequately sterilized dialysis, increase use of injected street drugs with unsterile needles.

With less than 1 percent of the population estimated to be HIV -positive, Egypt is a low-HIV-prevalence country. Unsafe behaviours among most-at-risk populations and limited condom use among the general population place Egypt at risk of a broader epidemic. According to the National AIDS Program (NAP), there were 1,155 people living with HIV/AIDS (PLWHA) in Egypt by the end of 2007 [12]. UNAIDs estimates for 2005 were higher, putting the number of HIV-positive Egyptians at 5,300 [13].

Egypt is a signatory to the 1988 UN Convention, as well as a party to the 1961 UN Single Convention on Narcotic Drugs and its 1972 Protocol, and the 1971 Convention on Psychotropic Substances [14]. Egyptian drug laws are strict, but enforcement is erratic. Penalties for drug trafficking are severe. Egypt is a minor player in the production of illicit narcotics and precursor drugs, but it is a major trans-shipment point for heroin from Afghanistan destined for Western Europe and North America. Domestic heroin and cocaine addiction is rising according to anecdotal evidence from both Egyptian law enforcement officials and medical professionals. Hashish is still the primary drug of choice in Egypt due to its low cost and availability [15,16]. Alcohol consumption is low and average per capita consumption is less than 0.1 L per year [17].

The household survey in 5 regions (Alexandria, Giza, Fayoum, Qalioubia and Ismailia) found a rate of 16% common mental disorders and 0.2% psychosis in people aged 18-64 [8]. This rate of CMD is consistent with rates found elsewhere (generally between 10-20%) while the rate of psychosis is a little low (generally between 0.5-1%) [5]. As elsewhere in predominantly Moslem countries, there is significant underreporting of suicides (according to the MOH statistics for 2008 the number of deaths due to suicide in the whole of Egypt is only 46 cases), because of stigma and traditional values which preclude discussion of what is a taboo subject. There is no formal statistical documentation of suicides in Egypt. All cases of overdose go to the Toxicity department and it is not recorded whether the toxicity episode was accidental or deliberate. Consideration is now being given to developing appropriate recording of suicides and a national suicide prevention strategy.

### Governance

Mental health services in Egypt are managed through two main systems, firstly the Mental Health Secretariat, headed by the General Secretary of Mental Health, which reports directly to the Minister of Health, and supervises 19 mental hospitals (6000 Beds); and secondly the Mental Health Departments in the General Hospitals (13 departments), with total bed capacity of about 121 beds. Also, there are 41 outpatient clinics present in some general and district hospitals. These departments and clinics are managed by curative care departments in the governorates, but responsibility for technical supervision and support has now also been handed to the MHS.

In addition, there are psychiatric departments in nine medical schools of public universities, comprising about 200 beds, and a further 750 beds in private hospitals. Thus, the Ministry of Health is by far the main provider of mental health services in Egypt [18].

There was no supporting policy development and implementation team to support the work programme of the General Secretary until 2007, when staff were appointed to coordinate various strands of policy work including primary care, specialist care and forensic services, and the health management information system. The MHS has also developed Directorates for Drug addiction, Child & elderly over the last 2 years. These Directorates are currently building up their human resources and have started to collaborate with international bodies to gain help with policy making e.g. Drug addiction is now a member of MedNET group. A service review of current Child Psychiatry services, including those dealing with ADHD, has been completed and an international Conference with workshops was held in 2010. There is a plan to develop a Directorate for Community Psychiatry within the MHS and in 2009 a feasibility study by the Italian Ministry of Foreign Affairs was completed to develop a pilot community psychiatric service 2010-11 near Alexandria.

There was a lack of systematic linkages between the MHS and other departments within MOHP, with other key ministries and key agencies, and therefore these linkages were developed in the mid 2000s, together with important links with the health sector reform programme.

Policy dialogue by the Egymen programme with the health sector reform programme ensured successful integration of mental health into the national health sector reform plan and general health policy, into the National Package of Essential Health Interventions, the Basic Benefit Package introduced in 2004, so that mental health appears as an integral component of health care at all levels, with defined interventions from primary care to tertiary hospitals. Consequently, the National Essential Drugs List outlines the type of psychotropic medicines to be provided from tertiary to primary care treatment settings, the national Health Management Information System includes mental health, and mental health has now been included in the Continuous Professional Development (CPD) programme for staff in primary care facilities. In addition to inclusion of mental health in the general health sector reforms, the Egymen project developed a national masterplan for mental health (the core components are summarised in Additional file 2) in 2004 to form the basis of formal mental health policy and support the overall Egyptian health policy. There were no national, governorate, or district mechanisms for governance, accountability and implementation of mental health policy, but this is now being addressed through the creation of the National Mental Health Commission - see below.

The 1944 mental health legislation was very outdated, and needed revision, and so new mental health legislation, first drafted by the Egymen project in 2005 was steered through the revision and consultation process by the MOHP and passed in 2009 [19]. Executive regulations for the legislation, first drafted as a code of practice in 2003 to introduce new concepts of human rights for wide consultation, and subsequently revised several times and disseminated for further consultation, were also passed in 2009. Work is now underway to consider the mental health relevant sections of other pieces of legislation with a view to updating and rationalising them.

The legislation includes the formation of a National Mental Health Commission (NMHC) which has now been established. Four subsidiary commissions will be formed, under the NMHC, to cover the whole country. The NMHC and its subsidiaries will not only oversee the implementation of the legislation, but also the implementation of national mental health policy, and will coordinate intersectoral liaison on mental health at the national level. General health management teams at governorate and district level were not orientated to mental health, did not have mental health representation and did not have mental health on their agenda or allocate resources to it, and this will also be addressed by the NMHC.

### Financing and cost benefit analysis

Service delivery is financed through taxation revenue. The funding of the health sector in Egypt comes mainly from the Ministry of Finance which funds services through budget transfers from the general revenues to the central government; then to the Ministry of Health. The budget of Mental Health Secretariat comes directly from the national revenue as part of the budget allocated to health. The MHS budget has increased from 80 Millions Egyptian pounds to 160 Millions over recent years, and is distributed based on the size, the needs and the development plans of each hospital. The budget for the mental health departments and outpatients departments comes directly from the hospital budget, which is a part of the curative care budget. Mental health expenditure in Egypt is no more than 2% of the total government expenditure on health. More than half of that allocation (about 59%) is spent on mental health hospitals.

There is substantial provision of unknown and probably variable quality by traditional health practitioners.

A preliminary economic cost benefit analysis was conducted of 58 current inpatients with schizophrenia in an acute ward of Al Abbassia Hospital in Cairo. The potential for discharge of this inpatient population was assessed and inpatient versus outpatient treatment costs was compared. The study estimated that 60% of inpatients could be immediately discharged on clinical grounds, and a further 10% were only constrained from discharge because they had long lost touch with their families. The study found that the cost of inpatient treatment was 29 LE per day compared with 9.7 LE for outpatient care. There are currently about 6000 patients with schizophrenia in inpatient facilities in Egypt. If the findings of this study are extrapolated to the whole schizophrenia inpatient group, it means that approximately 4,000 patients could be discharged and savings in excess of LE 28.6 Million could be realised. This is a substantial amount (the entire annual budget for Al Abbasseya hospital is LE 18 million). If the potential savings of LE 28.6 Million were entirely invested in family and community treatment of mental illness, approximately 3.1 million patient days could be funded in a budget neutral fashion for the government. This would provide almost 9,000 patient years of treatment in the community [Van Andel F: The Case for Economic Evaluation in Mental Health in Egypt - Preliminary Findings of a Cost-Minimisation Study in the Abbassia Hospital in Cairo - Egypt, submitted].

In practice, such redundant long stay beds are currently being used as acute beds, but it is planned that some hospital staff will soon move into community mental health teams. (See below).

The total contribution of the Finnish Government to the Mental Health Programme in Egypt for the period of January 2002 - June 2005 was EUR 2.450.000. The division between the main budget lines, according to the Programme Document, is presented in Table 1.

**Table 1:** Financial contribution of the Finnish Government to the Egypt mental health programme 2002-7

Budget line	2002-2005	2005-7
	EUROs	EUROs
Technical assistance	1.171.000 (48%)	534,000
Local technical assistance	168.000 (7%)	90,000
Training	340.000 (14%)	
Investments	290.000 (12%)	
Governance		240,000 (15%)
Integration MH to PHC		352,000 (22%)
Hospital Care		69,000 (4%)
MHIS		50,000 (3%)
Studies, monitoring, evaluation	50.000 (2%)	50,000
Operating costs	310.000 (13%)	125,000
Contingencies	121.000 (5%)	50,000 (5%)
**Total Finnish contribution**	**2.450.000 (100%)**	**125,000 (100%)**

The total contribution of the Finnish Government for the second phase of the Programme (During the period of July 2005 - December 2007) is EUR 1.590.000. The financial contribution of the Egyptian Government between 2002 and 2007 to the Mental Health Programme is set out in Table 2.

**Table 2:** The Financial Contribution of the Egyptian Ministry of Health to the Mental Health Programme 2002-7 (In Egyptian Pounds)

ITEM	2002-5	2005-7
**Office Space/Training Centers with their logistics**	445,000	297,000
**Human Resources (Mental Health secretary, Physicians, Nurses and other staff members)**	938,300	1,589,800
**Training**	108,380	1,704,520
**Total**	**1,731,680**	**3,591,320**

### Primary care

Egypt has a relatively well developed primary care system made up of two tiers, the first tier of which is family health unit (FHU), and the second tier is the family health centre (FHC). Each family health unit comprises doctor, nurse, social worker, and health educators, and each family health centre has a similar core team of doctor, nurse, social worker and health educator. In addition to this core team there are some specialists based in family health centres (e.g. paediatricians). In Alexandria, three family health centres have a psychiatrist visiting two days a week form the local mental hospital to run outpatient clinics within the FHC, and to liaise with the GPs over difficult cases.) The current role of the family health centre health team is largely to take referrals from the family health units, to see direct consultations, and to make referrals to the district level. Ideally they would also take responsibility of oversight to the catchment area population and the FHUs within the catchment area, but this is not yet a specified part of their role.

Until the Egymen Project, there was no continuing education for primary care staff on mental disorders, no good practice guidelines, and no antidepressants available in primary care, and no routine data collection on mental disorders. Since 2003, the Mental Health Programme in Egypt started a programme of CPD which was implemented for primary care staff (doctors, nurses, social workers and raeeda rafeya (health educators)) in five governorates. 138 trainers were trained, and over 3 years, 2193 primary care workers were trained, 654 physicians, 946 nurses, 593 social workers and health educators) and then supervised on a monthly basis. The project provided the primary care teams with basic, practical knowledge of mental health and the most common mental illnesses, and changed their negative attitudes towards mentally ill people and strengthened their positive attitudes. The trainees were encouraged to fight stigma in their communities. Their key role in prevention of mental ill health and promotion of mental health was emphasized. The importance of the whole PHC team in case management was discussed.

Since 2005, family health units where staff have been trained have collected mental health data, and there has been an expansion of the role of the nurses, social workers and health educators in primary care to include mental and neurological health work. There were no quality standards for mental and neurological health in primary care until the distribution of the Egypt adaptation of the WHO primary care guidelines. The training programme also initiated a programme of supportive liaison between primary and secondary care in the pilot governorates, with regular meetings to discuss criteria for referral, communication, shared care, guidelines, difficult cases etc.

### Specialist care

Evaluation of the status of mental health services in the country by the Ministry of Health in collaboration with the Egymen project confirmed that the country's health care system operates under extremely resource-restricted conditions, in terms of infrastructure, manpower and finances. Mental health specialist care is largely delivered at national level (national referral hospitals in Cairo and Alexandria) and at governorate level. (1-2 psychiatrists attached to each governorate hospital for around 3 million catchment population).

The total number of hospital beds for a population of over 75 million is 6156 (including the 680 forensic psychiatric patients at Khanka, 95 forensic beds at Abbassia, and 13 forensic beds at Ma'amoura). This is an average of less than 1 bed per 12,000 population across the country as a whole, compared with a WHO recommendation of 5-8 beds per 10,000 population (WHO 1996 World Health Organisation Recommendations for Mental Health Services, WHO, Geneva). In practice, when the national hospitals are excluded from the calculation, since it is not good practice to use them to admit people from a long way away from their communities, in most governorates there are only 20 beds per 3 M i.e. 1 bed per 150,000 population. With the prevalence of probable psychosis running at least 0.2%, [8]. It would be helpful to have psychiatric services available in every district as well as every governorate, and for every district hospital to have a 10-20 bed inpatient unit for brief admissions to assess and stabilise complex cases, as well as outpatient clinics. This would still leave the vast majority of psychosis cases to be managed at the health centre and dispensary levels.

There were 979 registered psychiatrists in 2009, including 285 consultant psychiatrists, the remainder classed as specialist psychiatrists. These figures have been increasing by around 6-9% a year. (These statistics are not entirely accurate because of emigration, temporary working in the Gulf countries, and also some university professors do not register themelseves as having consultant status but rather only use their professorial title.) Egypt has lost a high proportion of psychiatrists to rich countries [20]. 5% of all medical graduates go into psychiatry training, and 10% of all nurses. There were 1902 mental health nurses in 2006, 201 social workers and 77 psychologists. Specialists are mostly concentrated in the major urban centres, and so the specialist service for the other 30 governorates is largely delivered by 1 or 2 psychiatrists and a handful of psychiatric nurses for 3 million population. This lack of human resource and continued limited funding of mental health services, severely curtail access to specialist care. Nonetheless 25,443 out patients were seen in 2006.

The mental hospitals are institutional in design (e.g. Al Abbassia has about 2,000 beds) with large wards, little provision for personal possessions, patients are not allowed to wear their own clothes, there were no ward based activities and little opportunity for active rehabilitation. There was a striking lack of meaningful ward based activities, a lack of multi-axial assessments, care planning and regular case reviews, there were many long stay patients who could be rehabilitated. There was a lack of quality standards, good practice guidelines, psychosocial therapies and rehabilitation. In the last few years, the Egymen project established rehabilitation wards and intermediate services at Abbasseya and Khanka hospitals, where patients are given intensive rehabilitation and then discharged much earlier than would otherwise have been the case. As long stay patients are gradually rehabilitated and discharged, these long stay wards have been replaced with acute wards, largely serving the large Cairo population, and so the staffing requirement has to date remained the same.

Day centres have been established at Abbasseya, Heliopolis and Khanka hospitals. A small community service team has been established in Abbasseya hospital: this comprises of 2 doctors, 5 nurses, 2 social workers, currently serving 53 patients. The team members visit patients at home, follow them up, and give medication.

CMHTs are planned which will follow up and support patients in the community, and hopefully reduce the number of hospital admissions to hospitals. Some of the staff currently working with inpatient services will be transferred to work in the community. The first CMHT is now being developed in cooperation with an Italian donor.

Each governorate and some districts have psychiatric outpatient clinics. Outpatient clinics receive referrals from primary care and also directly. The outpatient services are not generally good at communicating the results of the referral back to the referrer or liaising and consulting with primary care on generic issues. Outpatient clinics do not always have enough medicines. Outpatient clinics do not generally offer psychosocial treatments.

There is a lack of systems for outreach to people with severe mental illness living at home, for home-based rehabilitation, and for intermediate services at governorate or district level. There are no community rehabilitation centres, day care centres or midway houses across the country apart from those linked to the national hospitals of Abbassia, Heliopolis and Khanka. When patients are discharged from hospital, there is a problem of them being unable to continue to access medicines. Although the mental health services in Aswan governorate are conducting useful outreach, enabling hospital admissions to be greatly reduced, as is a pilot outreach project at Abbasseya hospital.

Forensic services were very centralised at Abbasseya and Khanka, and needed updating. The Egymen project organised a group study tour to the UK followed by a 3 month placement in the UK for an Egyptian forensic psychiatrist, who has subsequently led forensic service development. There was a general lack of provision of child and adolescent services, services for the elderly, substance abuse, apart from some pilot activities for child psychiatry in Abbasseya (day care and outpatient departments), Khanka and Ma'amoura (outpatients), and so the Egymen project organised visits by Finnish experts to support subspeciality developments, which are now being y supported by the new Directorates for Drug Addiction, Children and Elderly in the MHS.

### Human resource development

Undergraduate medical training has not previously included adequate orientation either to mental health or to a public health population approach and work has started on updating the undergraduate curriculum, and the post basic primary care training. Primary care CPD is described above.

Most psychiatric training, with some exceptions, does not yet contain adequate orientation to service delivery to a defined population, to teaching skills, to liaison with primary care, to routine clinical audit, as well as to detailed multiaxial assessment and care planning, and to psychosocial treatments. Therefore, MHS reviewed the junior doctors' training programme in 2009. A National survey on trainer and trainee opinion on the current training programme was completed and a pilot appraisal system for junior doctors at Abbasseya Hospital was also completed, and efforts are now underway to improve the training programme.

Considerable efforts are now underway to encourage undergraduate medical students into psychiatry, including liaison with students' societies to improve the popular image of psychiatry, liaison with medical colleges to reduce stigma about psychiatry and maximise the potential for career development for psychiatrists. The psychiatry component of the undergraduate curriculum has been improved, the structure, content and delivery of the post graduate psychiatry training has been improved and salaries have been doubled for trainees working in Psychiatry at the MOH. Trainees are now encouraged to start their postgraduate training early, and are given protected training and learning time. They are encouraged to take the Egyptian Fellowship degree in Psychiatry. The Ministry of Health is establishing a recognized training programme in all districts; has piloted an appraisal system for trainees, is planning to generalize this appraisal system to all trainees, and is also planning to establish a CPD system for all psychiatrists including trainers.

General Nurses do not have adequate mental health in their basic training, they still have low status and lack generic skills to empower them to function in a multi disciplinary team. A capacity building programme for mental health nurses was developed by Egymen in 2004 in the 5 large mental hospitals. Nurses need to be oriented to psychosocial skills, rehabilitation, issues of patient welfare, including risk assessment and humane management of violence. 254 nurses received this training up to the end of the Egymen Project in 2007 and this capacity building programme for MH nurses has continued and now includes all 17 hospitals and centers under the MHS, so that in 2008 and 2009 another 250 nurses were trained, and 150 nurses were trained in patients rights in 2009.

Social workers do not have adequate mental health in their training. There is no occupational therapy training programme and other professionals lack an OT orientation. There are psychologists, but it is unclear how many are in the health sector, and what roles they are playing. Raeda Refeya are female community health workers with a high school education who are trained to do outreach activities, detect some diseases and provide health education.

There are no systematic mechanisms for delivery of CPD on mental and neurological health for other relevant public sector cadres including teachers, police, and prison staff. Traditional and religious healers are common and people regularly consult them either before or during consultation of orthodox services. There are no mechanisms for liaison or dialogue, there are some harmful practices and there are times when patients are referred late to the orthodox sector. There is a lack of evaluative research on health and social outcomes of traditional health practitioners, and on the possibilities of shared care.

### Intersectoral liaison

Before the project started, there was no systematic intersectoral liaison with social affairs, police, prisons, schools etc at national, governorate, district or primary care levels; no joint work programmes, joint agreements or trainings for key partners from other sectors; but this should now improve with the establishment of the NIMH and its subsidiaries. Specifically, there is still a lack of mental health training for social workers, police, prison staff and teachers. There are no prison mental health services. There are very few mental health NGOs and those which exist are mostly in the big cities, but are not well coordinated with each other for maximum effect, or in effective liaison with specialist services which remain largely unaware of NGO provision and activities. The Egyptian Association for Human Rights of Mentally Ill people is currently being formed and registered.

### Public education

Stigma is prevalent, with many misconceptions and lack of understanding about mental health. Until recently there has been no public mental health media campaign. A national media campaign was launched in 2006 by the EGYMEN project and further developed and continued by the MOHP, and involves TV, radio, posters for family health units and street advertisements [19].

## Discussion

Mental health services in Egypt, before the start of the programme, displayed a substantial emphasis on hospitals (mostly based in Cairo and Alexandria), with not enough attention to integrating mental health into primary care, resulting in inadequate prevention, early detection, prompt management, or community based aftercare; treatment and care, with not enough attention to rehabilitation and social inclusion; specialist expertise, with not enough attention to supporting other specialties to understand mental health issues, including family physicians; and on doctors, with not enough attention to developing the mental health skills and roles of other key cadres such as nurses and social workers. There was a concentration of mental health services and staff in hospitals in the largest cities, with insufficient decentralisation across the country to all governorates, districts and communities.

The project has achieved detailed situation appraisal, epidemiological needs assessment, inclusion of mental health into the health sector reform plan, essential package of health interventions, mental health policy guidelines to accompany the general health policy, adaptation of the WHO primary care guidelines, primary care training, construction of a quality system of roles and responsibilities, availability of medicines at primary care level, instigation of reforms in forensic services, rehabilitation and child psychiatry in Cairo and Alexandria, mental health legislation and Executive regulations. This project has worked closely with the MOHP (recently split into the Ministry of Health and the Ministry of Population) and other relevant ministries, and has sought to integrate its work systematically into the Egyptian system, so that the system can continue to function irrespective of donor funding or of personalities.

The strengths of this work have been the embedding of the Egymen project in the MOH, and the sustained commitment to mental health by the government of Egypt after the end of the bilateral aid programme. However, much remains to be done, including further decentralisation of mental health services in each of the governorates, intersectoral liaison at governorate and district level, and continuation of primary care training beyond the initial five pilot governorates.

## Conclusions

The project has demonstrated the importance of using a multi-faceted and comprehensive programme to promote sustainable system change. Key elements include a focus on the use of rapid appropriate treatment at primary care level, strengthening the referral system, encouraging intersectoral liaison, rehabilitation, social inclusion and public education. Egypt has declared its commitment to mental health and this project has provided a secure and sustainable policy foundation and sustainable implementation.

## Competing interests

NL is General Secretary for Mental Health Egyptian Ministry of Health, ES is Director of International Affairs, Mental Health Secretariat, AH was Project Manager for the Egymen Project, IS was a project officer for Egymen and RJ is Director of the WHO Collaborating Centre.

## Authors' contributions

RJ wrote the first draft of the paper and the other authors contributed equally to subsequent drafts. All authors read and approved the final manuscript

## Supplementary Material

Additional file 1 **Egypt Summary of Situation Appraisal**. Click here for file

Additional file 2 **Mental Health Programme in Egypt**. Click here for file

## References

[B1] Murray C Lopez AD The Global Burden of disease - a comprehensive assessment of mortality and disability from diseases, injuries and risk factors in 1990 and projected to 2020 1996 6 Boston Harvard University Press

[B2] Gureje O Jenkins R Mental health in development: re-emphasising the link The Lancet 2007 369 447 449 10.1016/S0140-6736(07)60211-6 17292748

[B3] Jenkins R McCulloch A Friedli L Parker C Developing a Mental Health Policy 2002 Psychology Press

[B4] World Health Organisation The world health report 2001. Mental health; new understanding, new hope 2001 Geneva; World Health Organisation

[B5] Institute of Medicine Neurological, Psychiatric, and Developmental Disorders; Meeting the Challenge in the Developing World 2001 Washington D C; National Academy Press 25057559

[B6] Jenkins R Gulbinat W Manderscheid R Baingana F Whiteford H Khandelwal S Deva M Lieh Mak F Baba A Townsend C The Mental Health Country Profile; background, design and use of a systematic method of appraisal. The International Consortium on Mental Health Policy and Services; objectives, design and project implementation International Review of Psychiatry 2004 16 31 47 10.1080/09540260310001635087 15276936

[B7] Country Profile http://www.mental-neurological-health.net

[B8] Ghanem M Gadellah M Meky F Mourad S El-Kholy G National Survey of Prevalence of Mental Disorders in Egypt-preliminary survey Eastern Mediterranean Health Journal 2009 15 65 75 19469428

[B9] CAPMAS Egypt Demographic Indicators 2008

[B10] World Bank World Development Indicators Database: Egypt 2009 http://ddp-ext.worldbank.org/ext/ddpreports/ViewSharedReport?&CF=&REPORT_ID=9147&REQUEST_TYPE=VIEWADVANCED

[B11] EL Zanatay F Way A Egyptian Demographic Survey and Health Survey 2008 2009 Cairo; El Zanatay and Associates, and Macro International

[B12] USAID Egypt final report. April 1999-September 2007 USAID's Implementing AIDS Prevention and Care (IMPACT) Project. Family Health International hwfoNrecw http://www.fhi.org/NR/rdonlyres/evj63cvzcgzmqvkjydpkzegt46wh43p65xfarmvxciobjqpq2ltr7hmjr7u4tzyhn37fhhqewlk3td/IMPACTFinalReportEgyptHV.pdf

[B13] UNAIDS World Health Organisation http://www.unaids.org/en/CountryResponses/Countries/egypt.asp. 2008

[B14] United Nations Office on Drugs and Crime http://www.unodc.org/unodc/search.html?q=egypt

[B15] U.S. Department of State International Narcotics Control Strategy Report 2008

[B16] United States Department of State Bureau for International Narcotics and Law Enforcement Affairs 1997

[B17] World Health Organisation EMRO Eastern Mediterranean Region country profiles; Egypt. WHO Global Status report on Alcohol 2004 2004 http://www.who.int/substance_abuse/publications/en/egypt.pdf

[B18] World Health Organization WHO-AIMS Report on Mental Health System in Egypt 2006

[B19] The Secretariat of Mental Health http://www.mentalhealthegypt.com

[B20] Jenkins R Kydd R Mullen P Thomson K Sculley J Kuper S Carroll J Gureje O Hatcher S Brownie S International Migration of Doctors, and Its Impact on Availability of Psychiatrists in Low and Middle Income Countries PLoS ONE 5 e9049 10.1371/journal.pone.0009049 20140216 PMC2816209

