# Supplementary material for: Mental health policy and development in Egypt - integrating mental health into health sector reforms 2001-9
Source: Int J Ment Health Syst. 2010 Jun 24;4:17. doi: 10.1186/1752-4458-4-17 (PMC2910029; doi:10.1186/1752-4458-4-17)
Supplement: Additional file 1 — Egypt Summary of Situation Appraisal. [file 1752-4458-4-17-S1.DOC]

**Additional File 1. Egypt Summary of Situation Appraisal (February 2003)**

| **Policy Component** | **Egypt’s’ situation** |
| --- | --- |
| **MENTAL HEALTH IN MINISTRY OF HEALTH** | general secretary of mental Health is responsible for mental health in MOHP,  enormous task for single person to do |
| Links between mental health division and other key divisions *inside* MoHP | e.g. Drug abuse, prevention and integration of mental health into PHC needs collaboration relevant MoHP divisions |
| Links *between* MoHP and other key ministries | need to develop close liaison at national level between the key ministries on mental health |
| Overall Governance of service delivery, continuing education and clinical supervision across Egypt. | need for national, governorate, district and family health centre mechanisms for governance for mental health, with appropriate integration to general health management structure. |
| Governorate mental health committee | need for governorate level management and oversight of mental health care, delivery of continuing education and delivery of clinical supervision in primary and secondary care in governorate |
| District mental health committee | need for district level management and oversight of mental health care, delivery of continuing education and delivery of clinical supervision in primary and secondary care in govern |
| Family health centre committee | need for FHC level management and oversight of mental health care, delivery of continuing education and delivery of clinical supervision in primary and secondary care in govern |
| Appraisal of context, needs, inputs, processes and outcomes at each level in service | Lack of detailed mapping at national and local levels |
| **PRIMARY CARE SERVICES** | |
| Family health centres and units | lack of continuing education on mental health for FHC staff |
| Good practice guidelines | need for good practice guidelines |
| Primary Health Care information system | PHC information system does not currently include mental health, but opportunity to insert as part of new health information system currently being piloted |
| PHC supply of medicines | inadequate supply of essential medicines to PHC. No antidepressants and probable use of diazepam for anxiety |
| PHC transport | limited transport for primary care staff to do outreach |
| PHC social workers | social workers in PHC |
| PHC health educators | health educators in PHC |
| PHC nurses | nurses in PHC |
| PHC quality standards | quality standards in PHC |
| PHC governance | no family health centre committees for mental health |
| Basic training | medical student training needs to be orientated to needs of PHC |
| Links between primary and secondary care | transport for districts to supervise FHCs is a major problem  ?? resource for regular communication  need agreed criteria for referral |
| **SECONDARY CARE** | |
| Governance | system of governance of service delivery, clinical supervision and continuing education is not working as well as it might |
| Basic training | see human resources section |
| Continuing education | need for system of trainers for continuing education  need for sustainable continuing education on regular basis |
| Activity programmes | ward activities very limited |
| Long stay patients | each in-patient unit has some long stay patients with inadequate rehabilitation |
| Admission assessment forms | lack of systematic assessments on admission |
| Care planning | lack of detailed individually tailored care planes which take account of physical, social, psychological needs. |
| Case reviews | no systematic regular multidisciplinary review of patients |
| Quality standards | lack of quality standards |
| Good practice guidelines | lack of good practice guidelines |
| Develop home based rehab. | need to facilitate home-based rehabilitation of severe mental disorders |
| Psychosocial therapies | no psychosocial therapies available |
| Medicines | medicines  need to access prescriptions without travelling long distances |
| Outreach | transport |
| Intermediate services at governorate level- | no intermediate services yet constructed |
| Intermediate services at district level | no intermediate services yet constructed |
| Intermediate services at FHC Level | None |
| District OPD | many districts don’t have OPD for mental health |
| Services for children and adolescents | lack of dedicated services for children and adolescents |
| **PUBLIC HEALTH EDUCATION** | need public health education at national, governorate, district, FHC and FHU levels |
| **HEALTH MANAGEMENT TEAMS** | ensure mental health is on the agenda of generic health committees at governorate, district and FHC level. |
| **TRADITIONAL HEALERS** | some interest in liaison; community participation in mental health promotion and care; no mental health referral guidelines; no liaison with traditional healers; no collaboration in managing CMI; no research on traditional psychotropic herbal medicines; no collaboration with international alternative therapeutic practises |
| **HEALTH INFORMATION SYSTEMS** | detailed info available in hospital admissions. Lack of meaningful integrated mental health information system covering the community and each level of care |
| **INTERSECTORAL LINKS** |  |
| Liaison with social welfare | need for links at national, governorate, district and FHC levels |
| Liaison with Police | police keen for liaison; lack of mental health information for police trainees; no governorate or district partnership with police in mental health care |
| Liaison with Prisons | no prisons mental health services |
| **PUBLIC HEALTH EDUCATION** | need public health education at national, governorate, district, FHC and FHU levels |
| Liaison with Health Education | limited resources for mental health programmes; School Health Projects focuses on health issues for schools; behaviour change is one of the key elements of substance abuse campaigns |
| Drug Abuse Control | substance abuse research is an integral component of multi-sectoral drug abuse control; no tobacco control legislation |
| **MENTAL HEALTH NGOS** | situation appraisal of current activities and roles of NGOs needed |
| **HUMAN RESOURCE DEVELOPMENT** |  |
| Psychiatrists | Too few psychiatrists |
| Nurses | Lack of empowerment of mental health nurses  consider extending PHC nurse role beyond filling in forms |
| Social workers | need to develop roles of social workers |
| Occupational therapists | no Occupational Therapy training to support mental health rehabilitation |
| **REFERRAL SYSTEM** | poor referral system between levels of care . No system for shared care. |
| **RESEARCH**  **CAPACITY** | insufficient research capacity at governorate, district and PHC levels |
| **MENTAL HEALTH**  **LEGISLATION** | existing mental health legislation is out dated |
| **REFUGEES** | 1 million near Sudanese border; no mental health programme, medicines, or programmes to reduce domestic violence. |
