# Supplementary material for: Mental health policy and development in Egypt - integrating mental health into health sector reforms 2001-9
Source: Int J Ment Health Syst. 2010 Jun 24;4:17. doi: 10.1186/1752-4458-4-17 (PMC2910029; doi:10.1186/1752-4458-4-17)
Supplement: Additional file 2 — Mental Health Programme in Egypt. [file 1752-4458-4-17-S2.DOC]

## Additional File 2. MENTAL HEALTH PROGRAMME IN EGYPT

**Objectives, activities, outputs, time frame, indicators, required resources and potential constraints.**

**May 2004**

| **Programme Component** | **Objectives** | **Activities** | Output | **Time frame** | **Monitorable Indicators** | **Resource required** | **Potential constraints** |
| --- | --- | --- | --- | --- | --- | --- | --- |
| **1. GOVERNANCE** | | | | | | | |
| **Policy team to ensure delivery of quality mental health services at all levels and sectors** | Support General Secretary for Mental Health with a multidisciplinary team to implement the programme below. | 1. Discuss with key persons on MOH 2. Find good candidates (could work part-time) | Effective team in post | Y1 | Team in post | Salaries and office in MOH | Unavailability of qualified personnel |
| **Links between mental health department and other key departments *inside* MOHP** | Strengthen links with key directorates and departments in MOHP | 1. Continue collaboration with Directorates/ Departments of Preventive services, Primary Care, Curative Services, Nursing, Technical Support administration, NHMIS, and Training. | Joint programmes of work | Y1-4  Cont. practice | Notes of meetings and joint activities | None | None |
| **Links with general health sector reform process** | Get mental health on agenda of Health Sector Reform process | 1. Ensure mental health representation on all MOH health sector reform meetings 2. Ensure mental health representation in all health sector reform documents | Mental health included in health sector reform | Y1 | Health Sector Reform Plan | None | Uneven distribution of mental health services |
| **Links with other health providers** | Strengthen links with other key health providers eg health insurance organization, private sector, army, universities | 1. Establish systematic linkages about service provision and training at national and governorate levels 2. Establish service level agreements eg between health and university for service provision and for training placements. |  | Y1-2 | Number of training courses  Universities participate in the conducted training |  |  |
| **Links *between* MOHP and other key ministries** | 1. Strengthen policy links with ministries of social welfare, education, housing, employment and media. 2. Support mental health division with national intersectoral mental health committee | 1. Establish national intersectoral mental health committee (NIMHC), Chaired by the General Secretary, to include representation from key parts of MOHP (eg health sector reform, health information, human resources), representation from other key ministries (eg education, social affairs, judges, religion, interior affairs (police/ prisons); representation from universities, key NGOs, (including user and family representation), and representation from each governorate. | National intersectoral mental health committee established | Y1-4 | Agenda and minutes of meetings | Budget for workshops and venues | Awareness about mental health problems, need for careful planning |
| **Programme Component** | Objectives | Activities | **Output** | **Time frame** | **Monitorable Indicators** | **Resource required** | **Potential constraint** |
| **Mental Health**  **Legislation** | Improve legislative support of human rights, care and treatment of people with mental illness. | 1. Consultation process to get community and professional views on new legislation 2. Draft and consult, and revise 3. Prepare new legislation 4. Develop Code of Practice 5. Training workshops for health professionals and all other sectors. 6. Implement Code of Practice | Revised legislation.  Code of practice. | Y1-4 | Legislation  Code of practice  Post for mental health lawyer | Budget for training workshops | Clarity of delegation responsibilities between different professionals |
| **Overall Governance of service delivery, continuing education and clinical supervision across Egypt.** | Establish and train national (see above), governorate, and district committees | 1. Start by establishing the governorate committees (GSMH attend each governorate committee at least once a year, districts to report to governorate committee once a year) 2. Establish the district committees when possible. Someone from governorate committee to attend each district committee once a year. The district committees should include representation from each PHC centre. 3. Each PHC can establish a local intersectoral forum eg with schools, police, social welfare, NGOs, user and family representatives, to discuss/ tackle local mental health issues of common concern, as well as to feed key issues up to the district committee. 4. Give specifically management/ public mental health training to the committees 5. Establish roles and responsibilities of each committee and annual work plans | Governorate, district and family health centre committees established | Y1 | Agenda and minutes | Travel,  Budget for training and meetings. | Current non availability of mental health services at district level.  Reduced levels of staff working in mental health. |
| **Governorate mental health committee** | Establish and train governorate mental health committee, and make appropriate links to general governorate health committee | 1. Agree membership for governorate intersectoral mental health committee (GIMHC) and the core health team. (The core health team are members of the GIMHC but are also directly accountable to the GMH, while the GIMHC as a whole is an advisory body which reports to the NIMHC) 2. Appoint each governorate committee. Membership include PHC, nursing, psychiatry, general health, health education, social welfare, police, prisons, religion (AWKAF), NGOs, (including user and carer representation), university, etc. 3. Organise training for each committee 4. Agree appropriate representation on governorate general health committee | Governorate mental health committee established and trained | Y1 | Evaluation of training | Travel,  Budget for training |  |

| **Programme Component** | Objectives | Activities | **Output** | | **Time frame** | **Monitorable Indicators** | **Resource required** | **Potential constraint** |
| --- | --- | --- | --- | --- | --- | --- | --- | --- |
| **District mental health committee** | Establish where possible district mental health committees and make appropriate links to general district health committees | Agree membership in principle for district intersectoral mental health committee (DIMHC) and for the core health team. (The core health team are members of the DIMHC but are also directly accountable to the GSoMH, while the DIMHC as a whole is an advisory body, which reports to the GIMHC). Membership will include primary health care, nursing, mental health, general health, health education, education, social welfare, police, prisons, religion (AWKAF), NGOs and university, employment.   1. Appoint each district committee 2. Organise training for each district committee 3. Agree appropriate representation from DIMHC to the district health committee 4. Send representation from DIMHC to GIMHC 5. (The DIMHC has capacity to form sub groups and to co-opt others as necessary for both consultation and to deliver the work programme) | District mental health committee established and trained | | Y1-2 | Agenda, minutes, evaluation of training | Travel,  Budget for training. | Current non availability of mental health staff at this level. |
| **Primary health care centre forum** | Where desired, each PHC can establish a local intersectoral forum eg with schools, police, and social welfare, NGOs, user and family representatives, to discuss and tackle local mental health issues of common concern, as well as to feed key primary-secondary care liaison issues up to the district committee | 1. Agree membership in principle 2. Agree appropriate representation on PHC general health committee 3. Send representation to district mental health committee | Primary health care centre forum established | | Y2 | Minutes, evaluation of training | Travel,  Budget for training. |  |
| **Monitoring-**  **(Appraisal of context, needs, inputs, processes and outcomes at each level in service)** | Compile national mental health country profile.  (More local situation appraisals to each of governorate, district and FHC committees) | 1. National country profile almost complete   *(Ask governorates, districts and FHCs to do something similar at local level)* | Detailed country profile available at national and local levels | | Y1 | Availability of national and local mental health profiles | Stationary | Needs annual upgrading |
| **Programme Component** | Objectives | Activities | **Output** | | **Time frame** | **Monitorable Indicators** | **Resource required** | **Potential constraint** |
| **2. PRIMARY CARE** | | | | | | | | |
| **Primary health care facilities** | Strengthen mental health at PHC level by  1.Develop continuing education for PHC staff  2. Where possible, as in Alexandria, place visiting psychiatrist in each FHC. | 1. Develop timetable and training programme for PHC staff 2. Train PHC staff in own right and train PHC staff to train PHCU staff | PHC training programme established PHC staff trained.  PHC knowledge and skills is regularly updated. | Y1-2 | | Curriculum  Evaluation of training. | Budget for training workshop | Shortage of psychiatrists  Availability of trainers to cover the whole country. |
| **Good practice guidelines** | Adapt, pilot and use WHO primary care guidelines | 1. Prepare draft guide to be piloted in the governorates 2. Pilot 3. Incorporate feedback and produce final version 4. Print and disseminate final version | Draft produced  Feedback from pilot  Final version printed and disseminated | Y1 | | Guide | Budget to print guidelines  Budget for orientation/ training  Travel | Guidelines not in use  Lack of encouragement |
| **Primary Health Care information system** | Add categories of mental disorder to family health unit information sheet | 1. Discussed with MOH, NIHCP 2. Pilot | HMIS contains 10 or more mental health categories | Y1 | | HMIS | Budget to pilot guidelines. |  |
| **PHC supply of medicines** | Ensure adequate supply of antidepressants and antipsychotics to PHC | 1. Add psychotropics to essential medicine list and primary care kits 2. Check distribution of psychotropics and re-ordering mechanisms | Primary care equipped with psychotropics | Y1-Y4  Contin. | | PHC records.  Prescriptions.  Pharmacy records. | Budget for medication and transport |  |
| **PHC transport** | Ensure PHC has access to transport for community outreach | 1. Put mental health on agenda for PHC transport | PHC transport used for mental health activities | Y1-4 | | PHC and Transport records | Transportation facility |  |
| **PHC social workers** | Develop their role to support mental health | 1. Include PHC social workers in FHU/FHC training 2. Include mental health in PHC social worker job plans. | PHC social workers trained.  PHC social worker job plans contain mental health | Y1-4 | | Numbers of PHC SWs trained.  Training evaluations.  Job plans containing mental health. | Budget for training | Unclear job description.  Small numbers  Misdistribution.  Lack of support and supervision |
| Programme Component | Objectives | Activities | **Output** | **Time frame** | | **Monitorable Indicators** | **Resource required** | **Potential constraint** |
| **PHC health educators** | Develop their role to support mental health | 1. Include in FHU/FHC training 2. Include mental health in PHC health educator job plans. | PHC health educators trained.  PHC health educators’ job plans contain mental health | Y1-4 | | Numbers of PHC health educators trained.  Training evaluations.  Job plans containing mental health | Budget for training | Unclear job description.  Small numbers.  Lack of support and supervision |
| **PHC nurses** | Develop their role to support mental health | 1. Include in FHU/FHC training. 2. Include mental health in PHC nurse job plans | PHC nurses trained.  PHC nurses’ job plans contain mental health |  | |  | Budget for training. | Unclear job description.  Small numbers  Lack of support and supervision |
| **PHC quality standards** | Improve quality of care in PHC  See also health management information system | 1. Develop quality standards for PHC | Quality standards produced | Y2 | | Quality indicators | Budget for printing |  |
| **PHC governance** | See general section on governance |  |  |  | |  |  |  |
| **Basic training of doctors for PHC** | Ensure medical student curriculum includes common mental disorders, psychosocial interviewing skills, orientation to PHC and Community. | 1. Link with universities. 2. Use existing good practice. 3. Insert key questions into exam 4. Establish placements for medical students in PHC | Curriculum revised | Y2 | | Curriculum  Exam questions  Evaluations of PHC placements for medical students |  | Lack of cooperation between ministry and university |
| **Basic training of nurses for PHC** | Ensure nurse curriculum includes common mental disorders, psychosocial interviewing skills, orientation to PHC and Community | 1. Link with nurse training colleges. 2. Use existing good practice. 3. Insert key questions into exam 4. Establish placements for nurses in PHC | Nurse curriculum revised | Y2 | | Curriculum  Exam questions  Evaluations of PHC placements for nurse students | Budget for workshops  Transportation | No unified curriculum for all universities  Lack of cooperation between PHC& nursing schools |
| **Programme Component** | Objectives | Activities | **Output** | **Time frame** | | **Monitorable Indicators** | **Resource required** | **Potential constraint** |
| **LINKS BETWEEN PRIMARY AND SECONDARY CARE** | | | | | | | | |
| **Liaison and support** | Establish supportive liaison between FHUs, FHCs and districts. | 1. Establish regular meetings between FHUs and FHCs, and between FHCs and districts *(see general section on governance)* | Regular meetings | Y1 | | Notes of meetings | Budget for meetings |  |
| **Transport** | Transport matrix be strengthened to support mental health supervision | 1. Ensure district mental health co-ordinator has access to transport to visit all FHCs regularly 2. Ensure FHCs have transport to visit their FHUs regularly | FHUs and FHCs receive regular visits | Y1 | | Transport records | Transport facilities | Unavailability of staff for supervision |
| **Communications** | Ensure means of speedy communication about individual patient care. | 1. Make available phones, fax, email, stamps, and hand held patient record. | FHUs / FHCs receive letter from specialists within 7 days of consultation | Y2 | | Patient records | Budget for equipment and distribution | Unavailability of clear referral system |
| **Referral System** | Strengthen referral process by developing clear procedures. | 1. Establish referral criteria 2. Develop forms for referral and forms for feedback and downward referral, or integrate substantial mental health section into general HSR referral forms | complex cases are referred for specialist care. Both specialists and PHC teams receive the information needed to enhance patient care | Y1 | | Referral forms  Outpatient diagnoses | Budget for forms. | Availability of trainers |
| 3. SECONDARY CARE | | | | | | | | |
| **Governance** | Strengthen governance of intersectoral mental health services to meet the needs of each governorate, taking into account the geography of different parts Egypt e.g. upper Egypt etc, and the availability of health care within them. | 1. Establish and train governorate and district committees, and develop their work programmes (see above) | Committees established and trained. Work programmes developed. | Y1-2 | | Committee membership.  Evaluation of training programme.  Work programme of each committee. | Budget for training | Availability of trainers |
| **Basic training** | Strengthen mental health component in basic training of doctors, nurses, social workers, village health workers, sanitarians etc (see human resources part) | 1. Review and revise mental health curriculum for medical students, nurses and other related health professions.  2. Ensure public health population perspective as well as individual clinical perspective. | Training made relevant to future population needs | Y2-3 | | Revised curriculum  Exam questions | Budget for printing and for orientation workshops | Each university has its own curriculum. |
| **Programme Component** | Objectives | Activities | **Output** | **Time frame** | | **Monitorable Indicators** | **Resource required** | **Potential constraint** |
| **Continuing education** | 1. Strengthen training capacity by developing teaching skills, management and facilitation education for governorate and district trainers 2. Strengthen and update skills of secondary care staff | 1. Training workshops for trainers 2. Organise strategic plan and deliver continuing education to secondary care staff, using trainers (see above) | Enhanced teacher skills  Enhanced skills in secondary care | Y1-4 | | Evaluation of TOT workshops on teaching skills  Evaluation of training | Budget for training  Budget for travel. | Not enough staff interested in training  Not enough supervisors |
| **District OPD** | Strengthen access to specialist referral close to home. | 1. Establish a mental health OPD clinic in each district. (In a few governorates, it will be possible to place a fulltime psychiatrist in each district. In most governorates, it will be necessary for the governorate psychiatrists to rotate their daily OPD to a different district each day to ensure coverage of all the districts in each governorate at least once every two weeks, thus bringing services closer to patients and facilitating local liaison with PHC. 2. Place on agenda of governorate mental health committee to allocate psychiatrists to district level to open OPD, and report progress to GSoMH | Local OPD opened in all districts | Y2-4 | | Audits of health care provision. | Budget to establish the service | Small numbers of psychiatrists |
| **District inpatient units** | Where possible, improve capacity to receive in patient care close to home | 1. In a few governorates, there will be sufficient human resource to establish small inpatient units at district level within general district hospitals. In most governorates, this approach will not be possible for many decades. | Local IPD opened in a few districts. | Y4 | | Audits of health care provision. | Budget to establish the service | Small numbers of psychiatrists |
| **Governorate Outpatient Department (OPD)** | Decentralise OPDs to district level-see above |  |  |  | |  |  |  |
| **Governorate Inpatient Departments (IPD)** | Establish small IPD in all general governorate hospitals, so that people do not need to transfer to the large mental hospitals a long way from home. | 1. Agree with Undersecretary of Health 2. Organise space and refurbish 3. Establish staffing levels 4. Establish procedures for admission, discharge, standards etc |  | Y1-4 | |  |  |  |
| **Large mental hospitals** | Review roles of large hospitals |  |  | Y1-2 | |  |  |  |
| **Programme Component** | Objectives | Activities | **Output** | **Time frame** | | **Monitorable Indicators** | **Resource required** | **Potential constraint** |
| Long stay patients | Review long stay cases in preparation for active rehabilitation. | 1. Ask all directors of hospitals to plan their audits of long stay patients 2. Audit all long stay in-patients to see who would be better off in rehab facility | Patients rehabilitated and able t go home | Y1-4 | | Patient progress reports and discharges of long stay patients. | Budget for rehabilitation | Not enough rehabilitation facilities  Lack of support for staff. |
| **Admission assessment forms** | Improve quality of assessments on admission | 1. Develop assessment forms for all admissions | Patient care improved through improved assessments | Y1-4 | | Patient assessment forms.  Quality of assessments | Budget for printing and training. |  |
| **Care planning** | Care planning improved. | 1. Develop multiaxial care planning form. | Patient care improved through improved care planning. | Y1-4 | | Care planning forms.  Quality of care planning. | Budget for printing and training. |  |
| **Case reviews** | Improve patient outcomes by increasing frequency and quality of case reviews. | 1. Develop system for regular case reviews | Improved outcomes and shorter length of stay through improved case reviews | Y1-4 | | Case review forms and case reviews. | Budget for training. |  |
| **Quality standards** | Improve patient outcomes by improved quality of care | Develop and implement quality standards for secondary care | Quality standards developed and disseminated | Y2-3 | | Quality standards.  Annual reports | Budget for orientation | Few supervisors Need to be simple rather than complex. |
| **Good practice guidelines** | Improve patient outcomes by use of good practice guidelines | Develop, pilot, disseminate good practice guidelines for specialist care of major disorders | Schizophrenia / depression guidelines drafted | Y1 | | Good practice guidelines | Budget for training and printing |  |
| **Psychosocial therapies** | Increase access to Psychosocial treatments | Insert psychosocial skills in basic training and continuing education | Psychosocial therapies included in curriculum | Y3-4 | | Curriculum | Budget for training. | Few psychologists and others with requisite skills to teach. |
| **Programme Component** | Objectives | Activities | **Output** | **Time frame** | | **Monitorable Indicators** | **Resource required** | **Potential constraint** |
| **Medicines** | 1. Increase availability of medicines in district and governorate IP and OP clinics 2. Increase access of discharged patients to medicines | 1. Review medicine availability in governorate and district hospitals and outpatients clinics 2. Review capacity of person discharged from hospital to access medicines | Medicine supply improved in hospitals and clinics and for people living at home | Y1-4 | | Prescription records  Client interviews | Budget for medicine and transport |  |
| **Inpatient ward activity programmes** | Improve level of ward activities to prevent institutionalisation*.* | 1. Prepare manual on ward activities for nurses. 2. Orientate nurses to need for varied daily programmes of ward activities   (Add activity programmes to standards of care for hospital settings)   1. Pilot standards of care in selected regions | Reduction of early institutionalisation.  Busy ward routines | Y1 | | Reduced length of stay. Improved patient outcomes | Budget for training | Nurses not orientated to ward activities. |
| **Develop home-based rehabilitation** | Consider how to develop home based rehabilitation, and whether it should be deployed for specialist care in collaboration with PHC | 1. Community based rehabilitation guidelines to be part of PHC and specialist training. | CBR guidelines drafted | Y3-4 | | Numbers of clients receiving home based CBR. | Budget for training and printing | Access to transport and staff |
| **Outreach** | Develop systems for outreach | 1. Mental health outreach plans should be part of the routine governorate and district outreach matrix that is integrated with other elements in PHC. 2. Develop outreach teams. 3. Transport for outreach, coordination, supervision, continuing education | Mental health professionals decentralised in their work schedules, and complex cases able to be seen in or near home. | Y2-4 | | Work schedules  Transport schedules | Budget for transport and training. | Small numbers of outreach staff |
| **Intermediate services at governorate level** | Patient outcomes improved by provision of intermediate services. | 1. Place on agenda of governorate mental health committee 2. Assess needs at governorate level 3. Build into forward budgetary planning 4. Find unused suitable buildings 5. Open dialogue with employers 6. Work out staffing | Intermediate services established | Y3-4 | | Audits of health care provision  Patient outcomes  Length of stay | Budget to develop intermediate service | Unavailability of suitable places |
| **Intermediate services at district level** | Review district needs and plan how to develop and fund | 1. Place on agenda of governorate mental health committee 2. Assess needs 3. Build into forward budgetary planning 4. Find unused suitable buildings 5. Open dialogue with employers 6. Work out staffing | Intermediate services established | Y3-4 | | Audits of health care provision  Patient outcomes  Length of stay | Budget to develop intermediate service | Unavailability of suitable places |
| **Programme Component** | Objectives | Activities | **Output** | **Time frame** | | **Monitorable Indicators** | **Resource required** | **Potential constraint** |
| **Intermediate services at PHC Level** | Ideally intermediate services would be distributed at this level, given the epidemiology, but that is probably long way in future, although individual FHCs may be able to liaise with the local community to establish social rehabilitation clubs | 1. PHC could start to consider their local needs for social and occupational rehabilitation, and supported accommodation (or intensive support to people living at home). | Intermediate services established | Y3-4 | | Audits of health care provision  Patient outcomes  Length of stay | Budget to develop intermediate services | Unavailability of suitable places |
| 4. TERTIARY CARE | | | | | | | | |
| **Forensic services** | Review needs for central and local forensic services, and improve outcomes for forensic patients |  |  | Y1-2 | |  |  |  |
| **Services for children & Adolescents** | Improve patient outcomes for children and adolescents | 1. Audit to assess need for C&A mental health services at governorate and district levels. 2. Consider referral Pathways for C&A 3. Form linkages with school doctors 4. Service developments 5. Implications for training 6. Develop guidelines 7. Develop community interventions, linking with NGOs, for children in need eg street children, employed children. | Improved specialist and intersectoral services for children and adolescents. | Y2-4 | | Child & Adolescent services  Patient outcomes | Budget for training and printing of guidelines | Small numbers of trainers |
| **Services for elderly people** | Improve patient outcomes for older people | 1. Audit to assess need special focus on elderly 2. Implications for training 3. Develop guidelines | Improved care of older people | Y2-4 | | Patient outcomes | Budget for training & printing of guidelines | Small numbers of trainers |
| **Liaison psychiatry** | Improve mental health outcomes of people with physical illness in general health care system |  |  |  | |  |  |  |
| **Drug Abuse Control** | Strengthen efforts to prevent and treat drug abuse | 1. Close liaison between mental health programme and drug programme on public education, epidemiology, family support, primary care guidelines and specialist services 2. Research should support drug abuse control plans | Drug abuse included in mental health work on public education etc | Y3-4 | | Campaigns, curriculum, guidelines. | Budget for research, training, printing and distribution |  |
| Programme Component | Objectives | Activities | **Output** | **Time frame** | | **Monitorable Indicators** | **Resource required** | **Potential constraint** |
| **Smoking** | Strengthen effort to prevent and treat nicotine addiction | 1. Increase availability of support for people wishing to stop smoking, especially children and adolescents as well as adults. |  | Y4 | |  |  |  |
| **REFERRAL SYSTEM** | Strengthen referral system from primary care to specialist care, and back again | 1. Develop agreed referral criteria for each level of care 2. Develop referral forms and forms for replies. 3. Develop shared care procedures 4. Pilot and review | Referral criteria  HS Reform referral and reply forms are adapted to include mental health  Shared care procedures | Y1 | | Forms  Procedures. | Budget for training, printing and follow up |  |
| **5.** **HEALTH MANAGEMENT TEAMS** | | | | | | | | |
| **HEALTH MANAGEMENT TEAMS** | Improve capacity of health management teams to address mental health. | 1. Place mental health as standing item on agenda of health management teams at governorate and district levels 2. Consider whether the mental health committees should be subcommittees of the general health committees and regularly report to them 3. Include mental health in any workshops for health management teams | Health management teams regularly have mental health on agenda | Y1-2 | | Minutes of meetings | Budget for training and meetings | The teams are overwhelm-ed by many activities. |
| **6. HEALTH INFORMATION SYSTEMS** | | | | | | | | |
| **HEALTH INFORMATION SYSTEMS** | There should be an integrated MH information system between dispensaries, health centres and OPDs in order to assist in shared care, calculations of needs for care, needs for essential medicines, and to support MOHP in its planning functions | 1. Health Management Information System to work closely with the mental health section to facilitate improved coverage of mental disorders by the HIMS. | HMIS includes mental health | Y1--4 | | HMIS records | Budget for printing forms, supplies and training. | HEALTH INFORMATION SYSTEMS |
| Programme Component | Objectives | Activities | **Output** | **Time frame** | | **Monitorable Indicators** | **Resource required** | **Potential constraint** |
| **7. Other Health Providers** | | | | | | | | |
| **TRADITIONAL HEALERS (referring to religious and other kinds of traditional healers)** | Improve mutual understanding between traditional and orthodox/public/state sectors in order to improve patient outcomes, reduce harmful practices and encourage early referral where this would improve patient outcomes. | 1. Collaboration with Traditional Medicine Section 2. Dialogue to reduce harmful practices | Harmful practices reduced | Y2-4 | | Client and family records | Budget to improve the skills of traditional healers | Resistance form the traditional healers.  Medical attitudes to traditional healers. (religious healers are accepted) |
|  | Improve detection and treatment of postnatal depression by collaboration with reproductive health programme for TBAs. | Mental Health Section to collaborate with Reproductive health and evaluate the feasibility  Training programmes for TBAs should include mental health | Increased treatment rates of PND | Y2-4 | | Training evaluations.  Treatment rates | Budget for training |  |
|  | Improve early referral of serious cases | 1. Develop referral guidelines 2. Give diagnostic algorithms to encourage referral of serious cases e.g. cerebral malaria, epilepsy, psychosis | Increased early referrals for THs | Y2-4 | | Patient records | Budget for training and printing |  |
|  | Assess scope for liaison | 1. Liaison is dependent on establishment of Traditional Medicine Legislation. | Improved opportunities for systematic liaison at national, governorate and district levels | Y1-4 | | Minutes of meetings | None |  |
|  | Enhance support given to chronic cases by operating shared care procedure with people living a long way from health professionals | Assess scope for shared care of chronic cases  Develop protocols for shared care  Audit practice and outcomes | Chronic complex cases receive intensive levels pf support despite living far from clinics | Y3-4 | | Patient records |  | Problems with accountability |
| **8. INTERSECTORAL LIAISON** | | | | | | | | |
| **Governance** | Strengthen systematic intersectoral liaison on mental health | See above sections on national, governorate and district intersectoral mental health committees |  |  | |  |  |  |
| **Programme Component** | Objectives | Activities | **Output** | **Time frame** | | **Monitorable Indicators** | **Resource required** | **Potential constraint** |
| **Liaison with social affairs** | Social outcomes for people with mental illness improved by strengthening liaison with social welfare. | 1. Visit ministry of social affairs 2. Visit local social welfare agencies 3. Develop joint committee working and joint work plans 4. Ensure doctors are familiarised with social welfare and roles. | Social welfare of people with mental illness improved. | Y1-4 | | Minutes and work plans | Transport |  |
| **Liaison with schools** | Mental health of children and adolescents in schools improved | 1. Liase with Ministry of education 2. Assess needs of teachers for training module about mental health issues 3. Mental health promotion in schools-see public health education 4. Assist education system to develop referral pathways for troubled children |  |  | |  |  |  |
| **Liaison with Police** | Quality of police role with people with mental illness improved. | 1. Joint meetings at national, governorate, district and PHC levels between health services and police 2. Ensure police follow good practice guidelines re handling people with mental illness 3. Negotiate with police authorities to include mental health issues in their training curriculum 4. Governorate and district MH training events about handling and documenting violent incidents could involve the police. 5. Ensure specialist services meet police needs for training in this area 6. Public education with media and lawyers about people's rights in relation to police and about mental health legislation. | Systematic liaison, good practice guidelines, training& public education.  Mental health modules included in police curriculum  Police invited to mental health trainings | Y4 | | Curriculum.  Training evaluations  Workshops. Participant lists. |  | Attitudes |
| **Liaison with Prisons** | Quality of care of people with mental illness in prison improved. | 1. Joint meetings at national, governorate, district and PHC levels between health services and prison service. 2. Ensure prisons follow good practice guidelines re handling people with mental illness 3. Organise training sessions for prison health care staff 4. Prison medical officers need better identification skills to direct people with mental illness to health facilities | Systematic liaison, good practice guidelines, and training, to ensure transfer of people with psychosis to hospital, and prevention/ treatment of common mental disorders | Y4 | | Minutes of meetings.  Records of transfers from prison to hospital.  Evaluations of trainings.  Psychotropics available in prisons. |  | Attitudes |
| **Programme Component** | Objectives | Activities | **Output** | **Time frame** | | **Monitorable Indicators** | **Resource required** | **Potential constraint** |
| **Religious Leaders** |  | 1. Liase with religious leaders at national, governorate and district levels. 2. Organise orientation/training seminars for the religious leaders |  | Y2-3 | | Minutes of meetings  Evaluations of trainings |  |  |
| **Liaison with universities** | University health sector collaboration improved to benefit of training, service delivery and health outcomes | 1. Liase with universities at the national level 2. Service level agreements with universities re training and service delivery |  | Y 2-3 | |  |  |  |
| **Liaison with employers** |  | 1. Liase with employers on prevention, treatment and rehabilitation in workplace 2. Work with employers to find job placements for people with severe mental illness |  | Y2-3 | |  |  |  |
| **9. PUBLIC HEALTH EDUCATION** | | | | | | | | |
| **PUBLIC HEALTH EDUCATION** | 1. Improve community awareness of mental health issues 2. Reduce stigma 3. Enhance mental health in all age groups 4. Liaison with schools | 1. Consider, develop and implement public health education possibilities/social marketing strategies in media, schools, campaigns, community groups, religious groups etc for children, adolescents, adults of working age and older people. 2. Find, stimulate and co-ordinate well known public figures to be product champions. 3. Establish mechanisms for briefing politicians at national and local levels. 4. Educate teachers to do mental health promotion in schools 5. Link to substance abuse and other risky behaviour education programmes | Pubic health campaigns.  Reduction of stigma and enhanced mental health at population level.  Mental health included in health education curriculum  Mental health education integrated with substance abuse/HIV prevention | Y2-4 | | Media reports of mental health.  Public attitude surveys.  School curriculum. | Budget to produce materials.  Post for PHE officer.  Budget for printing and developing campaigns.  Budget for training. | Difficulties in cooperation with different authorities.  Lack of public health education expertise.  Lack of support for teachers. |
| **Programme Component** | Objectives | Activities | **Output** | **Time frame** | | **Monitorable Indicators** | **Resource required** | **Potential constraint** |
| **10. NGOs** | | | | | | | | |
| **MENTAL HEALTH NGOS** | Strengthen NGO support of people with mental illness | 1. Governorate and district committees to map their NGOs 2. List NGOs in good practice guidelines 3. Visit of students/trainees to NGOs for orientation 4. Consider stimulating NGOs to play major role in intermediate services and in individual rehabilitation and work placements at governorate, district and PHC levels by stimulating psychosocial and occupational clubs. 5. Improve NGO cooperation | Map of NGOs.  Liaison with NGOs.  NGO roles enhanced. | Y1-4 | | List of NGOs available and in guidelines.  NGOs contribute to intermediate services. | Budget for training |  |
| **11. HUMAN RESOURCES**  Cooperation between different ministries responsible for HR, universities and training institutions | | | | | | | | |
| **Psychiatrists** | 1. Continue to train psychiatrists 2. Ensure orientation to community working, delivering a service to defined population, to support PHC,link with other sectors, to research | 1. Review postgraduate training of psychiatrists 2. Review continuing education 3. Add exposure to NGO developments, to PHC settings, to research and teaching skills, to population perspective and to community working, and to delivering a population to a service. | Revised curriculum | Y1-4 | | Curriculum.  Exams. | Budget for training and follow up |  |
| Nurses | Strengthen mental health nurses psychosocial skills and rehab skills  (Give PHC nurses mental health skills) | 1. Review basic training 2. Review continuing education 3. Access to guidelines and standards 4. Give some OT skills to nurses 5. Give all mental health professionals key psychosocial skills on rehab., relapse prevention etc | Revised training programmes. Guidelines disseminated to nurses. |  | | Curriculum.  Exams. | Budget for training and follow up |  |
| **Social workers** | Give social workers therapeutic skills, skills to achieve social inclusion of their clients | 1. Review basic training 2. Review continuing education at PHC and specialist levels | Revised training programmes and curricula |  | | Curricula  exams | Budget for training and follow up |  |
| **Occupational therapists** | Develop sustainable OT  *(In meantime ensure other professional have some basic OT skills)* | 1. Develop OT training programme 2. Include some OT skills into nurse training | OT courses, curricula |  | | Curricula, exams | Budget for training/ follow up |  |
| **Programme Component** | Objectives | Activities | **Output** | **Time frame** | | **Monitorable Indicators** | **Resource required** | **Potential constraint** |
| **Psychologists** | Strengthen their contribution to the mental health service. | 1. Audit where psychologists are currently employed and what they are doing outside and inside the health service. 2. Review basic training in mental health and orientation to community work and liaison between schools and PHC (with inclusion of dyslexia and mental retardation) 3. Strengthen cooperation with other mental health professionals 4. Develop psychologist posts at district level (and ideally at PHC level). | Psychologist courses and curricula | Y1-4 | | Curricula, exams, job plans at district and FHC level. |  | Negative attitudes of psychiatrists |
| **Raeda Refeza (RR)** | Develop their role to support mental health. | 1. Include mental health in job descriptions 2. Give training in mental health. | Training programmes.  Job plans in mental health. | Y1-4 | | Numbers of RRs trained in mental health.  Log books of people with mental health problems. | Budget for training and supervision | Lack of support.  Too short training. |
| **12. RESEARCH CAPACITY** | | | | | | | | |
| **RESEARCH**  **CAPACITY** | strengthen research capacity especially HSR and epidemiology | 1. Plan national epidemiological survey 2. Evaluate PHC training 3. Audit needs and outcomes of inpatients | Research used to support service improvements. | Y1-4 | | Research publications | Budget for conducting research |  |
